# Spectrum of MRI findings in central nervous system affection in Lyme neuroborreliosis

**DOI:** 10.1038/s41598-024-63006-x

**Published:** 2024-05-31

**Authors:** T. Volk, H. Urbach, V. Fingerle, J. Bardutzky, S. Rauer, Rick Dersch

**Affiliations:** 1 Clinic of Neurology and Neurophysiology, Medical Center – University of Freiburg, Faculty of Medicine, University of Freiburg, Breisacher Str. 64, 79106 Freiburg, Germany; 2 Department of Neuroradiology, Medical Center - University of Freiburg, Faculty of Medicine, University of Freiburg, Freiburg, Germany; 3 National Reference Center for Borrelia, Bavarian Health and Food Safety Authority, 85764 Oberschleissheim, Germany

**Keywords:** Lyme disease, Lyme neuroborreliosis, Cerebral MRI imaging, CSF, Neuroinfectious diseases, Encephalitis, Myelitis, Encephalomyelitis, Vasculitis, Neurology, Neurological disorders

## Abstract

Affections of the central nervous system (CNS) rarely occur in Lyme neuroborreliosis (LNB). CNS manifestations can have residual neurological symptoms despite antibiotic treatment. We explored the spectrum of CNS affections in patients with LNB in a tertiary care center in a region endemic for Lyme borreliosis. We retrospectively included patients treated at a tertiary care center from January 2020–December 2021 fulfilling the case criteria for LNB as stated in the current German guideline on LNB. Clinical data, cerebrospinal fluid (CSF) findings and MRI imaging were collected. We included 35 patients with LNB, 24 with early manifestations and 11 with CNS-LNB. CNS-LNB patients had encephalomyelitis (n = 6) or cerebral vasculitis (n = 5). Patients with early LNB and CNS-LNB differed regarding albumin CSF/serum quotient and total protein in CSF. Duration from onset of symptoms until diagnosis was statistically significantly longer in patients with encephalomyelitis. MRI findings were heterogeneous and showed longitudinal extensive myelitis, perimedullar leptomeningeal enhancement, pontomesencephalic lesions or cerebral vasculitis. CNS-LNB can present with a variety of clinical syndromes and MRI changes. No clear pattern of MRI findings in CNS-LNB could be identified. The role of MRI consists in ruling out other causes of neurological symptoms.

## Introduction

Incidence of Lyme borreliosis shows considerable variability in Europe^1^. In Germany, Lyme borreliosis occurs with an incidence of approximately 33/100.000 inhabitants, whereas Lyme neuroborreliosis (LNB) accounts for approximately 2.4% of these cases^2^. The most common manifestations of LNB are polyradiculoneuritis (Bannwarth’s syndrome) and meningitis^3^, often refered to as early LNB^4^. However, in about 7–14% of LNB patients, affections of the central nervous system parenchyma (CNS) can occur^5–7^. These manifestations are usually called late LNB, the incubation period however remains unknown in some of these patients. CNS manifestations of Lyme neuroborreliosis (LNB) include encephalomyelitis with spastic ataxic gait, micturition disorders and paraparesis^8,9^. Encephalitic symptoms with seizures, impaired consciousness or aphasia are rare, but have also been reported^10^. Another CNS manifestation is Borrelia-associated cerebral vasculitis. These patients develop cerebral ischemia and show abnormalities in intracranial cerebral vessels in angiographic imaging in addition to inflammatory cerebrospinal fluid (CSF) changes. A multifocal affection of medium-sized intracerebral arteries can be seen^11^. In peripheral LNB, histopathological studies show perivascular infiltration of lymphocytes in peripheral nerves via vasa vasorum without overt signs of vascular damage^12,13^. A similar mechanism could be present in intracranial vessels^14^. However, histopathological evidence in LNB patients with cerebral vasculitis is scarce^15^.

Course of disease in early LNB is usually favorable with antibiotic treatment^16,17^. However, as CNS-LNB is rare, dedicated studies regarding efficacy of antibiotic treatment and prognosis in these patients are lacking. Patients with CNS manifestations of LNB show more residual symptoms than patients with peripheral manifestations of LNB and may experience disturbing residual symptoms despite antibiotic treatment^10,16^. This higher rate of residual symptoms is probably due to persisting parenchymal damage. Literature on clinical spectrum and radiographic findings in patients with CNS manifestations of LNB is scarce^18,19^. Symptoms of CNS manifestations of LNB are not specific, and can be seen in other infectious and non-infectious conditions, potentially leading to a delayed diagnosis and deferred onset of antibiotic treatment in contrast to early LNB.

Previous studies show a variety of findings in magnetic resonance imaging (MRI) in patients with LNB, concluding that the primary role of imaging in LNB is to rule out other causes of neurological symptoms^18^. Despite some earlier reports, unspecific white matter hyperintense lesions are not more frequent in LNB patients than in healthy controls^20^. A recent study described a distinct ‘M-sign’ and a ‘tarsier sign’ respectively in five patients with brainstem encephalitis due to LNB^21^. Similar findings with hyperintensities in mesencephalon and pons are reported in another case of LNB with encephalitis^18^. We wanted to explore the spectrum of CNS affection in patients with LNB in a tertiary care center in a Lyme endemic region. A considerable emphasis was set on the spectrum of imaging abnormalities in CNS-LNB, as specific findings could guide further diagnostics and facilitate timely treatment.

## Methods

We screened electronic patient reports of a tertiary care center (Clinic of Neurology and Neurophysiology of the Medical Center—University of Freiburg) from January 2020 to December 2021 for patients with the diagnosis of LNB according to the case definition of the German guideline on LNB^22^. Patients with compatible neurological symptoms and positive anti-borrelial serology without further CSF testing are regarded as having ‘possible LNB’ and were excluded from further analysis. Anti-borrelial antibodies were assessed using a standard two-tier approach (ELISA followed by immunoblot)^14^. Patients with additional inflammatory changes in CSF (pleocytosis, quantitative intrathecal immunoglobuline synthesis for total IgG, IgA and IgM) are regarded as having ‘probable LNB’. Patients with additional evidence of intrathecal synthesis of anti-borrelial antibodies [Borrelia-specific antibody index (AI) ≥ 1.5^23^] or positive PCR or culture for *Borrelia burgdorferi* sensu lato are regarded as having ‘definite LNB’. Patients with concomitant tick-borne encephalitis (TBE) or other concomitant infections of the nervous system were excluded from further analysis. Demographical and clinical data were collected (age at onset of symptoms, sex, case definition, duration from onset of symptoms until diagnosis, CSF pleocytosis, CSF lactate, albumin CSF/serum quotient, CSF total protein, intrathecal immunoglobulin synthesis IgG/IgM/IgA). When available, measurements of C-X-C motif chemokine 13 (CXCL13) in CSF were collected. CXCL13 was measured at the German National Reference Center for Borrelia (VF) using ELISA from R&D Systems, Quantikine ELISA Human CXCL13/BLC/BCA-1. When available, data on PCR from CSF was collected. CSF was investigated using PCR for borrelial DNA. DNA extraction was performed using QIAGEN DNeasy®Blood &Tissue Kit (QIAGENGmbH, Hilden, Germany) according to the manufacturer’s instructions. For the detection of *B. burgdorferi* s.l. DNA two real-time PCRs were used, targeting the outer surface protein (OSP) A-gene and flagellin-gene (p41) as described elsewhere^24,25^.

According to clinical presentation, patients with polyradiculoneuritis, cranial nerve palsy or meningitis were regarded as ‘early LNB’, patients with signs of encephalomyelitis (e.g. spastic ataxic gait, micturition disorder, seizures) or cerebral ischemia due to vascular abnormalities as ‘CNS-LNB’. MRI abnormalities were retrieved as available from patient reports and reassessed by a senior neuroradiologist (HU) to evaluate the spectrum of imaging findings in CNS-LNB. Imaging findings will be presented separately in regard to the predominant clinical features in CNS-LNB (encephalitis, myelitis or cerebral vasculitis). Neurological outcome was evaluated according to the modified Rankin Scale (mRS) at the time of discharge from the hospital^26^. If a follow up visit was documented, mRS was used from the last available time point. The study protocol was approved by the ethics committee of the Medical Center, University of Freiburg (22-1299-S1-retro). The research was conducted in accordance to the Declaration of Helsinki. Written informed consent was obtained from all participants.

### Statistics

Statistical comparisons between two groups for non-parametric comparisons were performed with the two-tailed Mann–Whitney *U* test using exact *p* values. Comparisons for dichotomous data were performed with Fisher’s exact test. For comparison of multiple groups with non-normal distributed data, the Kruskal–Wallis test with Dunn’s post-test was used. A *p* value of < 0.05 was considered statistically significant. Statistics were performed with GraphPad Prism software Version 10.0.1 for Windows, GraphPad Software, San Diego, CA, USA, www.graphpad.com.

## Results

Over the time span of 2 years, we could identify 35 patients with probable or definite LNB, 24 with early manifestations and 11 with CNS-LNB. One additionally identified patient with CNS-LNB had concomitant TBE and was excluded from further analysis. Laboratory findings as well as demographical and clinical characteristics are shown in Table 1. All CNS-LNB cases fulfilled the criteria for definite LNB. Of these 11 patients with CNS-LNB, six had encephalomyelitis and five LNB-associated cerebral vasculitis. One patient with cerebral vasculitis had concomitant encephalomyelitis (Table 2: patient 7).

**Table 1:** Laboratory findings, demographical and clinical characteristics of included LNB patients.

	early LNB (n = 24)	CNS-LNB (n = 11)	*p* *v*alue
Case definition: definite n(%)^b^	14	11	0.001
Age in years (%)^a^	52.5 (18–69)	57 (18–56)	ns
Sex (F:M)^b^	10:14	7:4	ns
Duration from onset of symptoms—diagnosis (days)^a^	7.5 (0–90)	14 (0–730)	ns
CSF WBC count (/µL)^a^	90 (1–512)	155 (17–960)	ns
CSF total protein (mg/L)^a^	697.5 (180–2760)	1400 (587–9520)	0.006
Albumin quotient^a^	13.6 (3.2–39.3)	21 (9.1–116.9)	0.0092
Intrathecal quantitative immunoglobulin synthesis (IgG + IgA + IgM)^c^ (%)^b^	4 (16.6)	7 (63.6)	0.006
Intrathecal quantitative immunoglobulin synthesis (IgG + IgM)^c^ n (%)^b^	8 (33.3)	3 (27.3)	ns
Intrathecal quantitative immunoglobulin synthesis (IgM)^c^ n (%)^b^	1 (4.1)	1 (9)	ns
CSF lactate (mmol/L)^a^	2 (0.92–4.2)	2.26 (0.69–5.69)	ns
Anti-borrelial antibodies in Serum (IgG + IgM) n (%)^b^	14 (58.3)	6 (54.5)	ns
Anti-borrelial antibodies in Serum (IgG) n (%)^b^	5 (20.1)	4 (36.4)	ns
Anti-borrelial antibodies in Serum (IgM) n(%)^b^	3 (12.5)	0 (0)	ns
Positive anti-borrelial AI^c^ (IgG) n (%)^b^	14 (58)	11 (100)	0.015
Positive anti-borrelial AI^c^ (IgM): n (%)^b^	6 (25)	3 (27)	ns
Total positive anti-borrelial AI^c^ (IgG and/or IgM) n (%)^b^	15 (62)	11 (100)	0.033
CXCL13 (pg/mL)	n.a.^d^	1075 (4–12,820)	
PCR in CSF positive n (%)^b^	0 (0)	2 (20)^e^	ns
Modified Rankin Scale^a^	1 (0–2)	1 (0–3)	ns

^a^Median is shown with range in brackets for data with non-normal distribution. Statistical comparisons were performed with the Mann–Whitney *U* test.

^b^Comparisons for dichotomous data were performed with Fisher’s exact test.

^c^Assessed via Reiber’s formula^23^.

^d^Due to typical clinical manifestation, CXCL13 in CSF was not performed in patients with early LNB.

^e^One patient with encephalomyelitis, one with vasculitis.

**Table 2:** Clinical characteristics and MRI-findings in CNS-LNB patients.

Patient	Manifestation	Duration of symptoms until diagnosis (days)	Sex	Antibiotic treatment	CXCL13 in CSF (pg/mL)	MRI-findings	Outcome (mRS)
1	Encephalomyelitis	14	F	Ceftriaxone 16 days	1381	Bilateral T2-hyperintense mesencephalic lesions (Fig. 1A)	1
2	Encephalomyelitis	10	F	Ceftriaxone 13 days	12,820	Perimedullar and radicular leptomeningeal enhancement (Fig. 3B1)	3
3	Encephalomyelitis	300	M	Ceftriaxone 21 days	424	Perimedullar leptomeningeal enhancement (Fig. 3B2)	1
4	Encephalomyelitis	730	F	Ceftriaxone 14 days, followed by doxycycline 14 days	4630	T2-hyperintense lesion capsula interna and insula	1
5	Encephalomyelitis	14	M	Ceftriaxone 14 days, followed by doxycycline 7 days	1023	LETM (Fig. 3A)	0
6	Encephalomyelitis	90	M	Ceftriaxone 21 days	4	Tarsier-sign/rounded M-sign (Fig. 2A/B)	2
7	Cerebral vasculitis (concomittant encephalomyelitis)	90	F	Ceftriaxone 21 days	269	Basal leptomeningeal contrast enhancement, intracranial stenosis, vessel wall inflammation, LETM with hyperintense alterations of spinal grey matter (‘H’-sign) (Figs. 1B, 3C1/2, 4B)	1
8	Cerebral vasculitis	0	F	Ceftriaxone 21 days	1425	Cerebral ischemic lesion, intracranial stenosis and concomitant vessel wall inflammation (Fig. 4A1–4)	1
9	Cerebral vasculitis	1	F	Ceftriaxone 14 days	1665	Cerebral ischemic lesion, intracranial stenosis without vessel wall inflammation	1
10	Cerebral vasculitis	1	F	Ceftriaxone 21 days	413	Cerebral ischemic lesion, intracranial stenosis and concomittant vessel wall inflammation (Fig. 4C1–3)	1
11	Cerebral vasculitis	0	m	Ceftriaxone 21 days	1075	Cerebral ischemic lesion, intracranial stenosis, vessel wall inflammation	0

*mRS* modified Rankin scale, *LETM* longitudinal extensive transverse myelitis.

Due to the acute onset of symptoms, clinical and demographical characteristics of this patient were combined with cerebral vasculitis patients. In early LNB, 14 patients fulfilled criteria for definite LNB, 10 fulfilled criteria for probable LNB. CSF was collected before start of antibiotic treatment in all patients.

No statistically significant difference between early LNB and CNS-LNB was found regarding age, sex, CSF pleocytosis, and CSF lactate. CNS-LNB patients showed a statistically significantly higher amount of CSF total protein and a statistically significantly higher mean albumin CSF/serum quotient compared to patients with early LNB. Rate of positive AI for anti-borrelial IgG was statistically significantly higher in CNS-LNB than in early LNB. No difference was observed regarding AI for anti-borrelial IgM.

All but one case of CNS-LNB showed markedly elevated levels of CXCL13 in CSF. One patient with low levels of CXCL13 was treated with corticosteroids for several weeks before diagnosis of LNB was established. CXCL13 levels were not available from early LNB patients (Table 2: patient 6).

Median duration from onset of symptoms to diagnosis in early LNB was 7.5 days (range 0–90 days), in CNS-LNB the median duration from onset of symptoms to diagnosis was 14 days (range 0–730 days, *p* = 0.402). Regarding different manifestations of CNS-LNB, duration from onset of symptoms in patients with cerebral vasculitis (median 1 days, range 0–90) was statistically significantly lower compared to patients with encephalomyelitis (median 52 days, range 10–720, *p* = 0.035). Regarding early LNB and different manifestations of CNS-LNB, duration from onset of symptoms until diagnosis was statistically significantly shorter in early LNB compared to patients with encephalomyelitis (*p* = 0.046). Difference of duration of symptom regarding early LNB and cerebral vasculitis was not statistically significant (*p* = 0.511).

### MRI features

#### Early LNB

Of the early LNB patients, eight had cerebral MRI. In four of these patients, cerebral MRI showed unspecific white matter lesions, whereas two patients with facial palsy showed contrast-enhancement of the respective facial nerves. The other two patients showed no pathological findings in cerebral MRI. Spinal MRI was performed in three patients with early LNB, showing perimedullar and radicular contrast-enhancement in two cases and no pathological findings in one patient.

#### CNS-LNB

All patients with CNS-LNB had pathological findings in MRI imaging (either cerebral or spinal). MRI was performed an varying time points, but all in close proximity of diagnosis of LNB and beginning of antibiotic treatment. Exemplary MRI findings of individual patients are listed in Table 2 and are discussed in more detail in the following section with regard to the predominant clinical features.

#### Encephalitis

**Figure 1:**
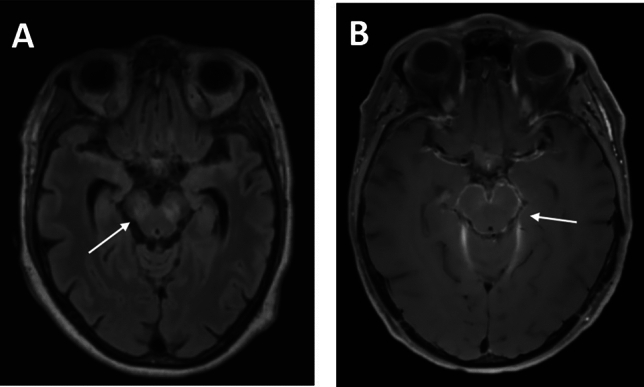
Cerebral MRI in CNS-LNB patients with encephalitis. (**A**) bilateral T2-hyperintense mesencephalic lesions in a patient with LNB encephalitis (T2 FLAIR). (**B**) leptomeningeal contrast enhancement around the cerebral pedunculi as a sign of basal meningitis as well as signs of vessel wall inflammation (T1w with contrast agent). White arrows depict representative findings.

One patient suffered from predominantly encephalitic symptoms with encephalopathy, tremor and disorientation (Fig. 1A, Table 2: patient 1). Cerebral MRI showed T2-hypertintense bilateral mesenecephalic lesions. One patient with encephalomyelitis and concomitant cerebral vasculitis showed leptomeningeal contrast enhancement around the cerebral pedunculi as a sign of basal meningitis (Fig. 1B, Table 2: patient 7).

**Figure 2:**
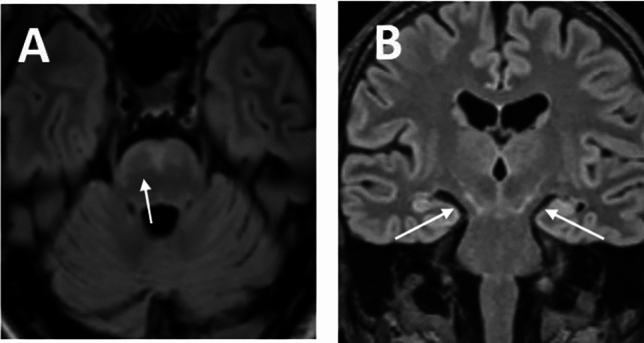
Cerebral MRI in CNS-LNB. (**A**) ‘Rounded M’-sign with T2-hyperintense pontine lesion in a patient with encephalomyelitis. (**B**) subtle ‘Tarsier-sign’ with T2-hypertintense mesencephalic lesion at the cerebral peduncles in the same patient. White arrows depict representative findings.

One patient showed the previously described rounded M-sign with concomitant tarsier sign with a symmetric pontomesencephalic T2-hyperintense lesion (Fig. 2, Table 2: patient 6).

#### Myelitis

**Figure 3:**
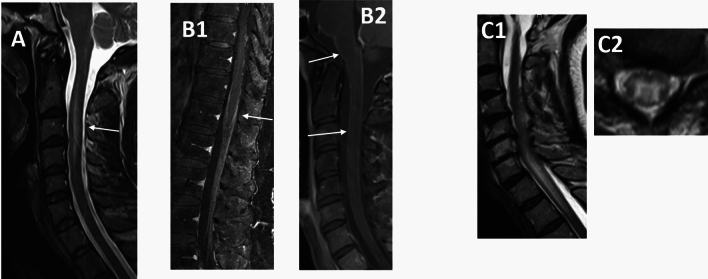
Spinal MRI in LNB patients with spinal affections. (**A**) longitudinal extensive transverse myelitis (LETM) of the cervical myelon (T2) (**B1/2**) perimedullar and radicular leptomeningeal enhancement (T1 fat sat with contrast agent) (**C**) LETM with hyperintense alterations of spinal grey matter (‘H’-sign) in a patient with concomitant CNS vasculitis (T2). White arrows depict representative findings.

Findings in spinal MRI in patients with myelitis were heterogeneous. We found longitudinal extensive myelitis (Fig. 3A, Table 2: patient 5) as well as perimedullar leptomeningeal enhancement (Fig. 3B, Table 2: patients 2 and 3). One patient with concomitant cerebral vasculitis and encephalomyelitis showed longitudinal extensive myelitis over 3 vertebral segments and hyperintense alterations of spinal grey matter (‘H’-sign, Fig. 3C, Table 2: patient 7). The extent of residual neurological symptoms showed no obvious correlation with the pattern of spinal involvement.

#### Cerebral vasculitis

All five patients with cerebral vasculitis had involvement of the anterior circulation, whereas two patients additionally showed involvement of the posterior circulation. No patient had isolated affection of the posterior circulation.

**Figure 4:**
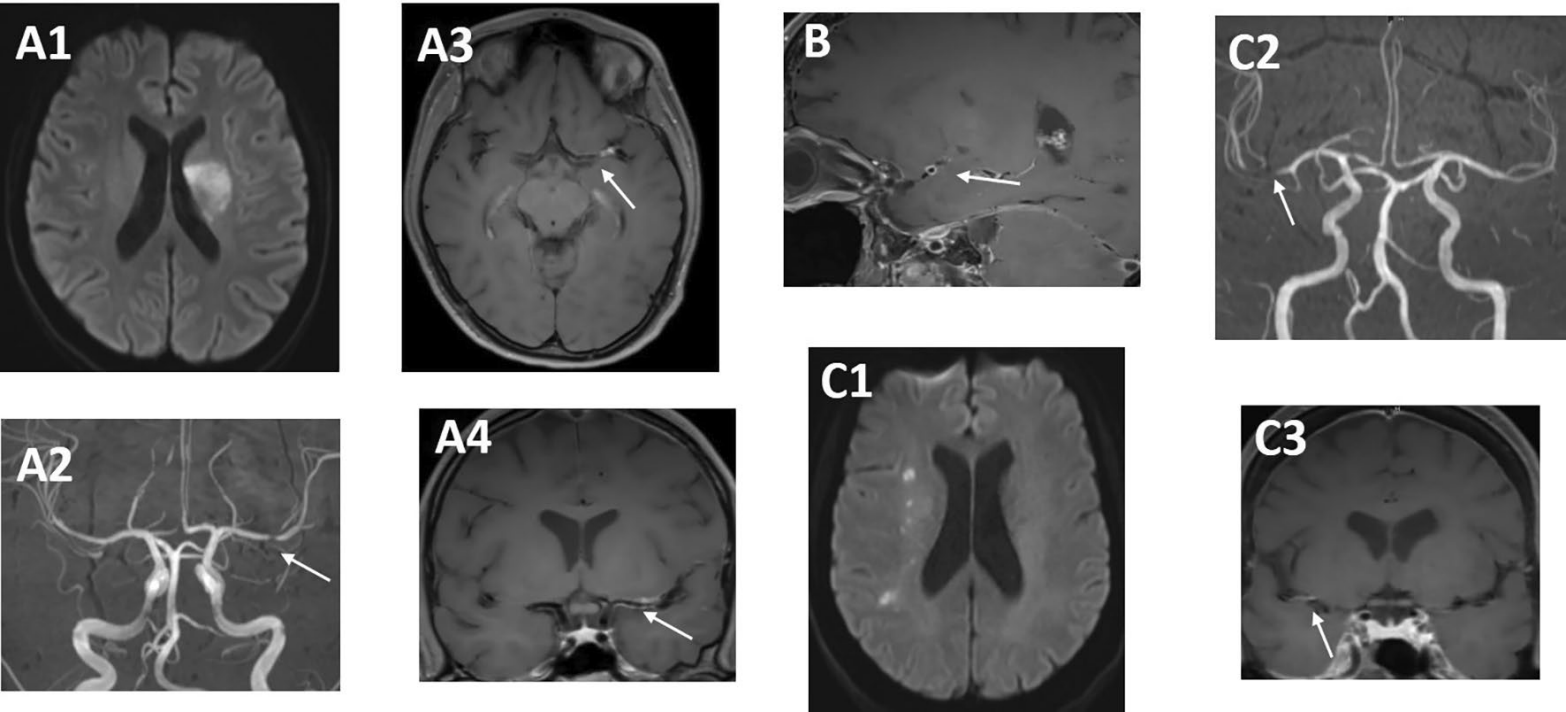
Cerebral MRI and MRI-angiography in CNS-LNB patients with cerebral vasculitis (Table 2, patient 8) (**A1**) ischemic lesion in the left MCA territory of the middle cerebral artery (MCA) (diffusion-weighted imaging). (**A2**) concomitant stenosis of the distal M1-segment as seen in the time-of-flight (TOF) angiography. (**A3/4**) correspondant contrast enhancement in vessel-wall imaging showing extensive vessel wall inflammation (T1w with contrast agent). (**B**) sagittal reconstruction showing concentric vessel-wall enhancement of the MCA in a patient with basal meningitis and vasculitis (Figure 1, T1w with contrast agent, Table 2: patient 7). (**C1**) scattered ischemic lesions in the right MCA territory (diffusion weighted imaging) (**C2**) concomitant stenosis of the distal M1-segment as seen in the time-of-flight (TOF) angiography. (**C3**) correspondant contrast enhancement in vessel-wall imaging showing local vessel wall inflammation (T1w with contrast agent, Table 2: patient 10). Each letters corresponds to one patient. White arrows depict representative findings.

MRI-angiography showed short-segmented stenosis of medium-sized cerebral arteries in all patients with cerebral vasculitis (exemplary Fig. 4, Table 2: patient 7–8, 10). All patients with cerebral vasculitis had vessel-wall-imaging, in 4 of these patients, long-segmented contrast-enhancement could be seen exceeding the extension of the stenosis seen in angiography sequences (e.g. Figure 4A, Table 2: patient 8). Parenchymal brain lesions were only seen in the context of cerebral vasculitis with concomitant cerebral ischemia (exemplary Fig. 4A1/C1, Table 2: patients 8–11).

### Treatment and outcome

Median duration of antibiotic treatment in CNS-LNB was 21 days (range 14–28). Treatment duration was statistically significantly longer in CNS-LNB compared to early LNB (*p* = 0.001). Patients with early LNB were treated with ceftriaxone (n = 9), doxycycline (n = 2) or a sequence of these two agents (n = 13). Duration of treatment was 14 days in 21 patients (ceftriaxone n = 8, doxycycline n = 1, sequential treatment n = 12). Extended treatment was performed in three patients (ceftriaxone 21 days n = 1, doxycycline 21 days n = 1, sequence 28 days n = 1) due to delayed resolution of symptoms. Median duration of antibiotic treatment in early LNB was 14 days (range 14–28). Patients with CNS-LNB were all treated with ceftriaxone, two patients with CNS-LNB had additional treatment with doxycycline after ceftriaxone (Table 2).

Of the patients with cerebral vasculitis, four were treated with methylprednisolone 1 mg/kg body weight for 2 weeks with oral taper over 2 months and additional platelet inhibition with long-term acetylsalicylic acid 100 mg p.o. The remaining patient with cerebral vasculitis had high-grade intracranial stenosis of the middle cerebral artery and was treated with 250 mg methylprednisolone for 5 days followed by methylprednisolone 1 mg/kg body weight with oral taper over 2 months as well as dual platelet inhibition for 3 months followed be long-tern acetylsalicylic acid 100 mg p.o.

Time to follow up was not standardized. Clinical data was restricted to patient records from initial clinical treatment and irregularly scheduled follow up visits. Median follow-up in CNS-LNB patients was 3.5 months (range 0.5–24 months). Patients with early LNB usually had no follow up visits, outcome assessment was therefore restricted to patient records from initial treatment at the last day of treatment. Therefore time to follow up in early LNB is statistically significantly shorter compared with CNS-LNB (*p* < 0.001).

Regarding neurological outcome after antibiotic treatment, early LNB patients did not differ from CNS-LNB patients (median mRS 1 in each group). However, CNS-LNB patients did show a broader spectrum of neurological outcomes (range 0–2 in early LNB, 0–3 in CNS-LNB; Table 1). However, the difference was not statistically significant.

In CNS-LNB patients, median mRS did not differ between patients with cerebral vasculitis and patients with encephalomyelitis (median mRS 1 in each group). However, patients with encephalomyelitis showed a broader range of persisting disability compared to patients with cerebral vasculitis, albeit showing no statistically significant difference (mRS range 0–3 compared to 0–1 respectively, ns.).

Residual symptoms in LNB consisted mostly on spastic-ataxic gait disorder; one patient with encephalomyelitis had to use a walker permanently.

## Discussion

Patients with CNS affection of LNB showed a variety of different patterns in cerebral and spinal MRI. In patients with myelitis, we could find longitudinal extensive myelitis as well as longitudinal leptomeningeal contrast enhancement. LNB should therefore be taken into consideration in patients presenting with these findings in spinal MRI. In cerebral MRI, CNS-LNB patients with encephalitis also showed a variety of findings with leptomeningeal contrast enhancement as well as mesencephalic lesions. The previously described rounded M-sign and tarsier-sign with characteristic symmetric T2 hyperintense pontomesencephalic lesions could only be identified in one patient with encephalomyelitis; the relevance of this finding should be further investigated^21^. CNS-LNB patients with cerebral vasculitis showed signs of intracranial stenosis and concomitant vessel wall inflammation. Otherwise, no specific lesion pattern could be identified in MRI. The number of CNS-LNB patients was limited, so we could not investigate if some MRI patterns are more frequent than others. Our results are in line with previous studies reporting heterogeneous findings in cerebral and spinal MRI in CNS-LNB patients^18^.

In early LNB, four patients showed unspecific white matter lesions, which are no specific finding in LNB^20^. Two patients with early LNB showed contrast-enhancement of the facial nerve in cerebral MRI and three patients with early LNB showed perimedullar and radicular contrast-enhancement, which is in line with previous reports and corresponding to the radicular inflammation^18^.

Regarding CSF analysis, no significant difference between CNS-LNB and early LNB could be found, apart from higher total protein and a more elevated albumin CSF/serum quotient in CNS-LNB possibly related to the pronounced parenchymal involvement and longer duration of symptoms and therefore longer duration of CNS inflammation.

In the study period, 31.4% of LNB patients had CNS-LNB, which is higher than expected from previous studies^7^. This is probably due to referral bias, as patients with more severe manifestations may be more prevalent in a tertiary care center. However, another explanation could be a shift in *Borrelia (B.) burgdorferi* species abundance in ticks^27^. *Borrelia garinii* and *Borrelia bavariensis* are more often associated with neurological manifestations of Lyme disease. If different strains of Borrelia are more likely to be associated with CNS-LNB is unkown. However, as PCR on CSF was negative in almost all patients, no certain conclusion can be made regarding specific *B. burgdorferi* species causing illness in our patients. Another explanation could be increased tick activity with associated higher virulence of *B. burgdorferi* due to changing climate factors^28^.

The finding of vascular long-segmented contrast-enhancement could corroborate the assumption of perivascular inflammation being the pathophysiological correlate of borrelia-associated cerebral vasculitis in contrast to direct vascular endothelial damage, as is also seen in peripheral nerve involvement^14^. Vessel-wall imaging sequences in cerebral MRI may be useful in assessing the extent of vascular inflammation in cases of LNB-associated cerebral vasculitis. All of our patients with cerebral vasculitis had involvement of the anterior circulation, with two patients additionally showing affection of the posterior circulation. This is in contrast with a previous study reporting predominantly affections of the posterior circulation^29^. However, a systematic review of 88 published cases with cerebrovascular manifestations of LNB showed a more even distribution of vascular territories (38% posterior circulation, 24% anterior circulation, 38% both)^11^. Therefore, LNB does not seem to affect a specific cerebral vascular territory preferentially and differences to previous studies appear to be random.

Follow up was not standardized. in early LNB disease outcome was assessed at the end of antibiotic treatment, in CNS-LNB follow up ranged from 2 weeks to 24 months, therefore data on disease outcome is biased. A considerable amount of patients with CNS-LNB showed residual neurological symptoms, albeit with varying degrees of disability. All cases with CNS-LNB showed considerable improvement after treatment. However, compared to patients with early LNB, who generally had an excellent outcome, CNS-LNB patients had a higher rate of residual symptoms within our follow-up period with a median of 3.5 months. This is in line with previous studies showing a relevant rate of residual symptoms on CNS-LNB^8,10^. On the other hand, patients with cerebral vasculitis due to CNS-LNB had an outcome similar to early LNB patients. Previous studies also reported a favorable outcome in patients with cerebral vasculitis due to CNS-LNB^11,30^. However, fatalities in cerebral vasculitis are reported in cases with extensive involvement of the posterior circulation^29^. Fortunately, we had no fatal cases in CNS-LNB, probably due to the limited involvement of the posterior circulation. None of our patients received a brain biopsy, which is regarded as reference standard for cerebral vasculitis. However, in cases with suggestive findings for vascular involvement in CNS imaging and a rapid response to antibiotic treatment invasive brain biopsy may be of limited additional value. Literature on treatment of LNB with cerebral vasculitis is scarce; in analogy to treatment of other forms of cerebral vasculitis we applied additional platelet inhibitors as well as corticosteroids^29^.

Duration from onset of symptoms until diagnosis and start of treatment was considerably longer in patients with CNS-LNB, especially in patients with encephalomyelitis. This shows that diagnosis of CNS-LNB is challenging and may be delayed in clinical practice due to other differential diagnoses. Especially in cases with unclear spinal symptoms, a lumbar puncture may be helpful to investigate treatable infectious causes.

All but one case of CNS-LNB showed highly elevated levels of CXCL13 in CSF, as was expected for this highly specific marker in the diagnosis of LNB^31,32^. The patient with low levels of CXCL13 was treated with corticosteroids for several weeks before diagnosis of LNB was established. Therefore CXCL13 expression in CSF could be reduced due to the immunosuppressive effects of corticosteroids. Only two cases of CNS-LNB and none of the early LNB cases showed a positive PCR in CSF, corroborating the limited use of PCR in the setting of LNB^33^.

## Conclusion

CNS-LNB can present with a variety of clinical syndromes and diverse changes in cerebral as well as spinal MRI. No clear pattern of MRI findings in CNS-LNB could be identified. MRI is more useful to rule out other causes of neurological symptoms than in substantiating a diagnosis of LNB.

## Data Availability

The data used to support the findings of this study are available from the corresponding author upon reasonable request.
